# An Ecological and Conservation Perspective on Advances in the Applied Virology of Zoonoses

**DOI:** 10.3390/v3040379

**Published:** 2011-04-15

**Authors:** Kurt J. Vandegrift, Nina Wale, Jonathan H. Epstein

**Affiliations:** 1 Center for Infectious Disease Dynamics, Pennsylvania State University, University Park, PA 16802, USA; 2 EcoHealth Alliance, 460 West 34th Street, New York, NY 10001, USA

**Keywords:** disease ecology, conservation, virology, vaccination, phylodynamics, metagenomics, endangered species, emerging disease, smart surveillance

## Abstract

The aim of this manuscript is to describe how modern advances in our knowledge of viruses and viral evolution can be applied to the fields of disease ecology and conservation. We review recent progress in virology and provide examples of how it is informing both empirical research in field ecology and applied conservation. We include a discussion of needed breakthroughs and ways to bridge communication gaps between the field and the lab. In an effort to foster this interdisciplinary effort, we have also included a table that lists the definitions of key terms. The importance of understanding the dynamics of zoonotic pathogens in their reservoir hosts is emphasized as a tool to both assess risk factors for spillover and to test hypotheses related to treatment and/or intervention strategies. In conclusion, we highlight the need for smart surveillance, viral discovery efforts and predictive modeling. A shift towards a predictive approach is necessary in today’s globalized society because, as the 2009 H1N1 pandemic demonstrated, identification post-emergence is often too late to prevent global spread. Integrating molecular virology and ecological techniques will allow for earlier recognition of potentially dangerous pathogens, ideally before they jump from wildlife reservoirs into human or livestock populations and cause serious public health or conservation issues.

## 1. Introduction

“A thing is right when it tends to preserve the integrity, stability, and beauty of the biotic community. It is wrong when it tends otherwise.”

—Aldo Leopold

Our planet is currently experiencing the sixth mass extinction event in history [1]. Although there are problems with estimating the total number of extant animal species [2], recent extinction rates are thought to be 100- to 1000-times greater than past rates determined from the fossil record. It is estimated that as many as 140,000 species are perishing each year [3]. Although habitat loss and fragmentation are the main drivers of this high extinction rate, infectious disease also contributes to animal population declines either independently, by reducing population size, or through interactions with other processes [4,5].

Indeed, these anthropogenic changes to habitat are also contributing to a second biological crisis: an increase in the rate of emerging and re-emerging infectious diseases (EIDs) [6,7]. Of these EIDs, 75% are **zoonotic** (see Table 1 for a definition) [8] and 37% are RNA viruses [9]. The high mutation rate of RNA viruses coupled with their ability to recombine and reassort allows for a rapid rate of evolution. In turn, this makes them highly adaptable and thus able to both exploit the new hosts and habitats afforded by a changing environment, as well as to develop resistance to treatments [10]. Examples of zoonotic RNA viruses which have emerged relatively recently include SARS coronavirus (SARS CoV), West Nile virus (WNV), Chikungunya virus, the 2009 influenza A (H1N1) and human immunodeficiency virus type 1 (HIV-1) and 2 (HIV-2). Cumulatively, these ailments have claimed hundreds of millions of human lives and cost the global economy hundreds of billions of US dollars. Among all viruses, HIV-1 causes the greatest amount of human mortality [11] and its immunosuppressive nature is facilitating the resurgence of “old” pathogens (*i.e.*, tuberculosis) in human populations [12]. It is possible that the emergence of HIV is also encouraging cross-species transmission and that these disease threats may have a severe, negative impact on wild animal populations [13]. A multi-disciplinary approach will be necessary to combat the crises of extinction and disease emergence because human, ecosystem and animal health are inextricably linked. In recent years, great advances have been made in virology and disease ecology, particularly towards elucidating the mechanisms behind the emergence and evolution of zoonotic viruses. Thus, it is important to review and consider how advances in virology and disease ecology complement each other.

The roots of ecology date back to Theophrastus in the 4th century B.C. [14]. The concept of food chains originated in the 17th century and Darwin and Wallace put forth the theory of evolution in the 18th century [15], but ecology did not become a prominent field until 1927 when two key advances transformed the study into a proper discipline. In this year, Charles Elton published his *Animal Ecology* [16] and Kirmack and McKendrick (1927) [17] formulated a model to describe the progress of an epidemic in a homogenous population [16,17]. In the 1960s, Rachael Carson’s *Silent Spring* generated concern for the environment and thrust ecologists into a new political field where preserving the integrity of our global ecosystems was the priority [18]. Even so, the Society for Conservation Biology was not established until 1985 [19]. As a part of this transition, ecology shifted from a descriptive science to one of prediction, reflecting the hope that ecologists might mitigate changes which can have negative impacts upon the ecosystem. Ecologists have branched out into the study of parasites and disease as it has become increasingly apparent that parasites are inextricably linked to the ecology of their hosts and environments, to the point where they have been a driving force in the evolution of sexual reproduction and in the shaping of biodiversity [20,21]. Over the past 30 years, disease ecologists have developed the study of parasites and pathogens in the wild. This knowledge has been synthesized into mathematical models which describe the dynamic properties of ecosystems and predict how parasites and pathogens flow through them. [22,23]. These models are becoming more commonly integrated into epidemiological studies that seek to predict outbreaks or periods of time when cross-species spillover risk is highest.

Parallel to this progress, the field of virology, particularly the subfields of molecular virology and viral evolution, have also been burgeoning, largely due to advances in technology that have made molecular assays and genetic sequencing more accessible to a greater number of scientists. The development of high-throughput sequencing has greatly increased our ability to efficiently detect known viruses as well as to discover new types of viruses, thereby improving our understanding of viral diversity, pathology and evolution. This increased capacity has spawned the development of new fields of study. For example, phylodynamics allows researchers to determine the origin of circulating viruses in space and time. Mutations among viral strains can be used to investigate interactions among host species as well as long-range host movement via corridors and flyways. Phylodynamic analyses can also inform livestock management practices, as was the case with Foot and Mouth disease in the United Kingdom [24].

Conducting viral surveillance in animal **reservoirs** and invertebrate vectors can help explain circulation within host species; observed patterns of zoonotic transmission; and even allow for the prediction of periods of increased risk of zoonotic transmission (e.g., Rift valley fever and rainfall [25]; West Nile virus (WNV) and American robin (*Turdus turdus*) migration [26]; as well as hantavirus in mice [27,28]). Understanding viral ecology in wildlife reservoirs and identifying high-risk human-wildlife interfaces is especially critical in the context of ever increasing globalization, whereby transportation networks facilitate rapid spread of pathogens well beyond bounds where traditional epidemiological methods can be effective [29–31]. The 2009 influenza A (H1N1) pandemic spread from the presumptive point of emergence in La Gloria Mexico to New Zealand in just under a month [32] while SARS radiated from Guangdong, China to 26 different countries within several months [29].

The negative impacts of emerging infectious diseases are not limited to humans. Indeed, wildlife conservationists have documented several mass mortality events in other animal species. Western lowland gorillas (*Gorilla gorilla gorilla*) have been decimated by Ebola virus [33] and an especially virulent calicivirus, rabbit hemorrhagic disease virus, spread through both domestic and wild rabbit populations, resulting in tens of millions of deaths [34]. In some instances the viruses have **attenuated**, while in others the animal populations have been brought to the brink of extinction. Importantly, the risk from disease to humans and animals should not be separated. The global transportation network facilitated the introduction of infected vectors (e.g., mosquitoes) into New York and WNV caused both avian and human mortality, and this virus has subsequently spread across the United States [26].

Globalization, host ecology, host-virus dynamics, climate change, and anthropogenic landscape changes all contribute to the complexity of zoonotic viral emergence and disease, and create significant conservation and public health challenges. Comprehensive and collaborative scientific approaches that transcend disciplinary boundaries are necessary to address these challenges. It is the goal of this paper to review new methods for understanding viral dynamics and illustrate how and when these techniques can be used by not only public health officials, but also disease ecologists and conservation biologists.

## 2. Phylodynamics

**Figure 1.**
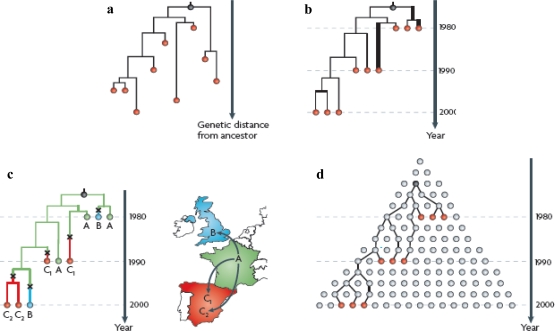
Phylodynamic techniques. (**a**) A rooted molecular **phylogeny** represents the evolutionary relationships between individual viral sequences, as represented by circles. These phylogenies *may* reflect transmission chains, however sampling must be sufficient for them to do so, while **recombination** may obscure ‘true’ relationships between viral sequences. (**b**) Simple **molecular clock** theory, predicated on the neutral theory of molecular evolution [48] assumes that mutation occurs at a constant rate over time, thus the time that has elapsed since a pair of virus strains diverged from a common ancestor may be quantified. Methods that account for differences in the evolutionary rates of different strains, and for variation in these rates through time, have been recently developed [49]. Here variants represented by thick lines evolve much faster than those represented by thin lines. (**c**) Using a phylogeographic approach, the location at which a sequence was sampled may be mapped onto the viral phylogeny and the likely spreading trajectory of the virus inferred. While parsimony approaches have been popular, powerful Bayesian methods that account for uncertainty of dispersal process and historical phylogeny have been developed to reconstruct viral dispersal events [44]. Crosses on the phylogeny represent such viral dispersal events, in this example. (**d**) Coalescent theory provides the basis for many phylodynamic approaches. Here, circles on the same row represent temporally simultaneous infections. Working back from sampled infections (red circles), lineages can be traced back to the most recent common ancestor (black circle) via hypothetical, unsampled ancestors (grey circles). The time it takes for sampled lineages to coalesce is dependent on a variety of variables (*i.e.*, viral **effective population size**, **population structure**, selection, stochastic infection die-out and recombination). A variety of methods are available to test for selection and recombination. Figure and legend adapted with permission from Macmillan Publishers Ltd: Nature (Pybus, O.G., Rambaut, A. Evolutionary analysis of the dynamics of viral infectious disease), copyright (2009) [50].

The phylodynamic paradigm, established in 2004 [37], exemplifies the power of a multidisciplinary approach. It unites the ecological and evolutionary study of viruses and builds upon advances in sequencing technologies and **coalescent theory**, by which gene genealogies are reconstructed backward in time [38]. The analysis of phylogenetic trees enables researchers to address many of the primary questions posed by disease ecologists (Figure 1). In some cases this approach can provide an estimate of a virus’s **basic reproductive number** (R_0_), which is a measure of parasite fitness [39]. Phylodynamics has far-reaching applications for the control of viruses in both human and animal populations, in addition to being vital to our understanding of the interconnectedness between them.

Phylodynamic studies can be used to identify reservoir species as well as defining the spatial and temporal origin of emerging infectious diseases [37]. They can also help to elucidate how these viruses spread following their emergence [39]. Firstly, chains of viral transmission can be extrapolated from the branching topology of phylogenetic trees. One example of the utility of this approach is with rabies virus. Rabies causes thousands of human deaths a year in Africa and has been implicated in the decline and local extinction of several populations of African wild dog (*Lycaon pictus*) [40,41]. Lembo *et al.* analyzed sequences of rabies virus from the Serengeti, revealing that domestic dogs were the reservoir of the virus and that they had transmitted it to other resident carnivore populations on repeated occasions [42]. This work has applicability in that it can be used to design efficient and effective vaccination strategies, both to alleviate current distress and prevent future outbreaks [40]. Secondly, by mapping the geographical origin of each sequence onto the nodes of phylogenetic trees, the geographical origin of a virus might be identified. Wallace *et al*. (2007) [43] used this phylogeographic approach to identify Guangdong province, China as the most parsimonious origin of highly pathogenic H5N1 strain of avian influenza and to delineate the most likely pathways of viral spread [43]. However, a recent Bayesian analysis of this data did not support the conclusion that H5N1 had dispersed from Guangdong to Indonesia [44]. Instead, the Bayesian analysis suggested it had spread to Indonesia from Guangxi or Hunan in China. This example demonstrates that different statistical techniques may yield different conclusions. As yet, neither the Bayesian nor the frequentist method is universally considered to be superior and there is much room for improvement as statistical **phylogeography** develops as a field. Phylogeographic tools have also been applied by Walsh *et al*. (2005) [45] to locate the putative origin of the Zaire strain of Ebola virus. In an attempt to resolve the controversy over the time of emergence and spreading trajectory of Ebola in the Congo Basin, they then used spatial data and two different tests for the impact of selection on the virus genome [45]. Where a virus is expanding in range, as the Zaire strain of Ebola virus appears to be, using a ‘landscape genetics’ approach may help identify geographical barriers to viral spread and help identify vulnerable human or wildlife populations lying in the path of infection [46,47].

Phylodynamic analyses are not without limitations. Dense and representative sampling at a scale equivalent to epidemiological surveys is required to fulfill the potential of phylodynamics for understanding epidemics of rapidly-evolving viruses [51]. The construction of phylogenetic trees from these viral sequences may be complicated by **recombination** and, in the case of segmented viruses, **reassortment** of viral genomes [36]. As a result of these processes, the genes on a single viral sequence may have very different origins (see the discussion of the different origins of the hemagglutinin and neuraminidase segments of H5N1 avian influenza in Lemey *et al.* 2009 [44]). Therefore, concatenated analysis of multiple genes may be confounded. The ability to construct phylogenies is further limited by the total viral genetic information available. GenBank is a vast public database that contains records of genetic sequences; however, its usefulness is dependent upon the willingness and/or ability of individuals and organizations to submit viral sequences. Governments and industrial institutions may be reluctant to report sequences of economically important viruses (*i.e.*, avian influenza) due to the potential negative economic impacts that may ensue. Although phylodynamics is currently encumbered by the aforementioned factors, there is hope for progress. Advancements in coalescent theory will help us to deal with the phylogeny construction problems caused by recombination and reassortment. They will also facilitate better utilization of genomic and spatial data, provided these advancements are also accompanied by a simultaneous increase in computing power, which is also currently limiting.

### Inferring Host Population Structure and Recent Demography from Viruses

The utility of phylodynamics is not limited to questions of interest to virologists and disease ecologists. This approach may also inform investigations of host population biology and, in so doing, aid in the development of conservation policy. Host molecular markers (e.g., microsatellites, mitochondrial DNA) are used by conservation biologists and ecologists to infer population structure, historical **demography** and other critical features of wildlife populations [52] and have proved particularly powerful when analyzed in combination (*i.e.*, [53,54]). Recently, it has been demonstrated that the pathogens of host populations might also be useful to this end. Research using helminths and bacteria has revealed patterns of ancient human migration and dispersal [55,56] identified ancient refuges of rodent and bird taxa [57,58] and shown that there was past contact between contemporary non-sympatric bat species [59]. However, there have been few attempts to utilize viruses to this end (save [60,61]).

It is surprising that viruses have not been used more for the inference of host population biology since some of their characteristics make them ideal for doing so. Most viruses have large population sizes and short generation times, and many replicate using a highly error-prone RNA-dependent RNA polymerase, causing them to accumulate many more mutations (nucleotide changes) per unit time than the host genomes [39,62]. Consequently, viruses may provide information about host demographics on a shorter timescale than molecular markers of the host. One of the signature tools of phylodynamics, the **Bayesian skyline plot**, might also be utilized to infer changes in historical population size of the host. These plots incorporate the use of a molecular clock and coalescent theory to infer historical changes in virus population sizes without assuming a predefined demographic model [63]. It is important, however, that the timescale over which the evolutionary dynamics of the virus population can be reliably reconstructed is appropriate for the parameters of interest in the host population.

Unfortunately, the very characteristics that make viruses useful for estimating host population structure and demography may also impede the analyses. Multiple substitutions can occur quickly in the viral genome and this will obscure the host population’s actual evolutionary history. Meanwhile, variations in the transmission mechanisms of viruses (vertical *vs.* horizontal) can alter the ability to accurately infer a virus’ relationship to a host population. Cross-species transmission is also problematic in that it can cause pathogen phylogenies to inaccurately reflect the history of their hosts [62]. Therefore, before the genetic information contained within a virus population can be used to infer the population structure and demography of the host, it is critical to test for congruence in the evolutionary history of the host and virus populations. This is accomplished by statistically comparing the respective phylogenies within the relevant timescale. Viruses with high host specificity have a greater likelihood of exhibiting such congruence.

Feline immunodeficiency virus (FIV) is known for high host specificity and is, thus far, the only virus to have been used to elucidate changes in host population structure and size. From a phylogenetic analysis of FIVpco, the FIV type specific to the cougar (*Puma concolor*), Biek, Drummond, and Poss (2006) [61] inferred that the North American population of cougars became subdivided during the last century but subsequently expanded in both size and range. Subsequently, Antunes *et al*. (2008) [60] used the distribution of FIV_Ple_ subtypes in the Serengeti to infer that recent **admixture** has occurred between the region’s lion (*Panthera leo)* populations. These recent changes in felid population size and structure could not have been inferred from host genetic data.

There is great potential for the further use of this technique by conservation biologists and ecologists, and for it to complement existing methods which utilize host genetic data. Host genetic markers are used to define management units for conservation purposes [64]. Many ‘flagship’ endangered species have long **life histories** [65], a feature that correlates with both extinction risk in certain regions [66] and difficulty in reconstructing recent demographic history from molecular markers. Because of the latter, the use of viral genetics to define management units may be an important avenue of exploration. In addition to aiding the definition of management units, viral data could be used to analyze the consequences of management activities and other environmental changes on target species. Where viral genetic diversity exists in a spatially heterogeneous distribution, viral movement patterns could be used to study the migratory behavior of animals (as macroparasites have been [67,68]). As such, researchers could monitor the use of wildlife corridors and the efficacy of control measures aimed at limiting the range of a host species [69,70]. Where viruses can be readily amplified from non-invasively collected samples (see [71]), the above objectives could be achieved in a cost effective manner with minimal disturbance of the study species. Viruses with specific transmission routes may also serve as proxies for behaviors related to transmission (*i.e.*, sexually transmitted diseases). Similarly, where cross-species transmission occurs, viruses might be indicative of types of sustained, direct contact between different, sympatric taxa which facilitate such transmission, for example predator-prey interactions [72]. At the broader ecosystem level, inferences about long-term evolutionary processes might also be made by examining the phylogeographic structure of numerous host and virus populations of a region (see [73]).

## 3. Viral Discovery and Metagenomics

A major hurdle for both virologists and ecologists is defining the biodiversity of life. At present, scientists do not know the actual number of mammal species, much less the diversity of viruses they harbor [2]. Indeed, the diversity of viruses known to infect the house mouse (*Mus musculus*), a staple in biomedical research, is not yet completely known. Recent advances in genetic sequencing, including high-throughput sequencing and other “next-generation sequencing” techniques, as well as MassTag PCR and microarray multiplex assays [74,75] have made the study of microbial diversity feasible [76]. These technologies have facilitated a movement from classical virology, where the focus was on disease etiology, toward a broader discipline that considers the rest of the viral diversity or “the virosphere”.

**Metagenomic** studies have used next-generation sequencing to study biodiversity in substrates such as ocean water and soil [77,78]. Metagenomics has also been used to screen human and animal clinical samples in order to determine etiologic agents of disease or to describe the microbial flora normally present in a vertebrate host—in many cases the result has been the discovery both of novel pathogens and novel associations between clinical disease and agent [79–82]. As technology becomes more affordable, and thus accessible, there will be increasing opportunities to ask large-scale questions such as: How does the virosphere vary across space and time? How does it vary across species? Can we use this to define risk of cross-species transmission or to inform conservation efforts? And how might co-infections with these undiscovered viruses influence the dynamics of the more well known viruses?

The paucity of information about viral diversity within a host poses problems for research progress. It is difficult to understand viral pathogenesis and transmission without completely understanding the dynamics of co-infections. Indeed, it is currently difficult to ascribe a host’s symptoms to an individual virus with any certainty because a virus’ actions, and even its ability to infect the host, could be a function of another (possibly undetected) co-habitant of the host. Evidence of interactions between co-infecting species has been clearly demonstrated [83,84] and it will be critically important to elucidate the interactions that occur between multiple pathogens as well as the combined effects they may have on a host’s immune system. Broadening our understanding of the diversity of pathogens that exist in human and animal hosts through wildlife and domestic animal surveillance will significantly improve our ability to recognize novel zoonotic agents in the context of a disease outbreak. Phylogenetic information obtained from comparative sequence analyses can improve our understanding of the impact of sequence mutation on virulence, as well as inform decisions about vaccine development. A final noteworthy benefit of viral discovery efforts is that these techniques should be important for identifying candidates for future vaccines as a virus’s most worthy competitor is often another virus.

## 4. Vaccination

From a health perspective, vaccination is arguably the most important technology that has arisen from the study of viruses. Vaccination offers a direct means of intervening in a host-pathogen system and it has become routine in many parts of the world. Efforts to this end have resulted in the eradication and/or control of smallpox, polio, mumps, measles, rubella and most recently, rinderpest.

Vaccines take several forms including live-attenuated viruses; inactivated whole viruses; inactivated toxins and viral protein subunits, and these are often delivered in combination. While live-attenuated vaccines have been predominant, a new generation of techniques including gene delivery and nano-technologies are being used to develop highly-efficacious and safer vaccines, that have less risk of **reversion** [85]. New types of administration methods are also being developed with oral, aerosolized and nasal vaccines currently on the market. These less invasive administration techniques decrease labor costs associated with administration and offer increased capacity for mass-dispersal of vaccines to both humans and free-ranging wildlife [86].

Vaccination campaigns aimed at both protecting threatened species and decreasing public health risks via animal vaccination have taken place. Swiss health officials were pioneers in this field, using oral vaccines to control rabies in wild red foxes (*Vulpes vulpes*) [87,88]. These vaccines were inserted into chicken heads which were distributed in the wild beginning in 1978 [88]. As can be observed from the supplemental movie (Video S1), their initial barrier approach evolved into a large-scale treatment of infected areas and resulted in rabies being successfully pushed back to and then eliminated from the Swiss Alps [87,88]. Following the success of these trials, campaigns were conducted in Western Europe [89] and Canada [90] with similar results, though the situation in the United States has proven more challenging. Other successful vaccination examples include canine distemper virus in black-footed ferrets (*Mustela nigripes*; [91]) and Ethiopian wolves (*Canis simensis*; [92]), as well as rabies in Florida panthers (*Felis concolor coryi*; [93]) and African wild dogs [94,95]. A caveat to this success is that there is growing evidence that vaccination against a specific strain of pathogen can result in inadvertent selection for related co-infecting strains. Thus vaccination can influence the dynamics of a pathogen [96].

The possibility of inadvertent viral strain selection highlights the importance of understanding the long-term evolutionary and ecological consequences of vaccination. Indeed, where threatened or endangered animals are concerned, mishaps may prove disastrous. Attenuated canine distemper vaccines did not provide immunity to critically endangered black-footed ferrets, while the use of a live canine distemper virus vaccine resulted in clinical distemper arising in one of the few remaining populations [97]. Ideally, long-term clinical trials with suitable animal models might avert these problems. These trials should also be used to provide an *a priori* understanding of how vaccination might shape future evolutionary processes. In contrast to the ferret experience, efforts with the endangered Ethiopian wolf serve as an example of a successful vaccination program. Wolf populations were suffering severe mortality due to rabies and distemper acquired from the wild dogs that shared their home range [98]. On the basis of a spatially explicit individual-based model, which indicated rabies could be controlled in dogs given just over 60% coverage [99], Knobel *et al.* executed an intensive vaccination plan [92]. Both the extent and duration of outbreaks in the treated areas were limited and, although monitoring and continued vaccination are required, the situation appeared to be under control in 2008 [92].

Wildlife vaccination campaigns are also being investigated as tools to limit public health risks. Tsao *et al.* vaccinated mice in an effort to break the cycle of Lyme disease and reduce the risk of emergence in human populations, in which it causes tens of thousands of deaths per year in the US [59,100]. In the same vein, Griffing *et al.* [101] have tested the efficacy of vaccinating American robins to interrupt the WNV transmission cycle. This species can absorb up to 75% of the potentially infective mosquito bites in early spring and is thus a key host in the WNV system [102]. Targeted vaccination of this single species could potentially result in herd immunity and reduce the risk of human infection as well as decreasing wildlife mortality. These works exemplify how ecological knowledge can be used to identify and exploit some of the heterogeneities which so often dominate the dynamics of pathogens.

While the lasting efficacy of wildlife vaccination efforts has yet to be demonstrated with either endangered species or in breaking the transmission cycle of human pathogens, an increasing number of researchers are drawing attention to systems where it seems feasible [99,103]; demonstrating that intricate knowledge of host and virus ecology can greatly reduce the amount of vaccine coverage that is necessary to control these viruses.

The problems entailed by the sheer number of viruses, viral resistance, the explosive potential for spread, and the economic burden, make it clear that currently available vaccination methods do not provide a sustainable solution for either human or animal disease. The unambiguous indication is that researchers need to work towards the goal of developing a predictive framework where risk can be defined for different scenarios and not only to rank pathogens, and species, but also, places and times of year that can be identified as more or less precarious for global health. Pending questions include: Which geographic areas will experience more disease and conservation problems? Which areas pose the highest risk for pandemic spread of pathogens? What characteristics of hosts and viruses make them more or less likely to be involved in cross-species transmission events? And what are the relative roles of genetic relatedness and contact rate for transmission? Some modeling work and reviews of historic data have been informative [104,105], but novel uses of phylogenies of both viruses and hosts (as discussed above) provide promise for progress to this end, especially when coupled with high quality surveillance data. Once we have this information, scientists will be able to design “smart surveillance” strategies whereby valuable vaccine resources can be efficiently targeted and efficiently distributed.

## 5. Reservoir Host Ecology

Ecological studies can effectively inform conservation as well as public health policy. Gaining knowledge of reservoir host ecology can be critical for the development of eradication strategies. Most viral disease systems are dominated by heterogeneities and identifying and understanding these can be crucially important when trying to interrupt the chain of events that leads to persistence. Ecological studies of WNV have shown how forest fragmentation and decreased biodiversity can alter transmission among avian hosts as well as to humans [106]. Likewise, researchers have used satellite imagery to identify habitat characteristics that accurately predict the prevalence of Sin Nombre virus [107,108], a hantavirus that uses the Deer mouse (*Peromyscus maniculatus*) as a reservoir host and it occasionally infects and kills humans [107]. These studies epitomize the type of effort scientists will need to successfully fight viral pathogens in the future. However, piecing together emerging disease and conservation problems *ex posto facto* is only of limited value. Increased pathogen surveillance and ecosystem process monitoring may provide the insight necessary to mitigate problems before they become serious human health or conservation concerns. This is especially the case for zoonotic viral pathogens where the reservoir hosts are known and a targeted approach is feasible.

Rodents rank as the number one reservoir of emerging and re-emerging zoonotic viruses [9]. Conveniently, these small mammals also present a manageable system for studying disease dynamics [109–111]. Individuals can be marked and sampled individually through time. Their locations as well as their contacts with other individuals can be measured. As such, wild populations of rodents can be valuable as model disease systems to address relevant questions like: Are there key hosts for transmission? How does prevalence vary seasonally or over time? What is the contact rate between the reservoir and humans? How do these pathogens flow through populations? The answers are of critical importance because they provide an indication of when and where there is increased risk of a zoonotic event whereby a human becomes infected, or when a species becomes at genuine risk of extinction. By monitoring and manipulating wild populations, one might also be able to identify factors that may increase a pathogens chance of emerging. For instance, what characteristics of hosts and viruses make them more or less likely to be involved in cross-species transmission? And what are the relative roles of genetic relatedness and contact rate for transmission? Long-term monitoring and surveillance in reservoirs will also enlighten us to the kind of **aggregations** and other heterogeneities that exist through time and that and can be exploited with efficient vaccination campaigns.

## 6. Conclusion

We are experiencing a global increase in the rate of emerging viral zoonoses, which are primarily driven by anthropogenic activities such as land-use change, agricultural intensification, and driven by global travel and trade [112]. In order to adequately understand, predict and ultimately interrupt the processes by which zoonoses cross the species barrier from their natural reservoirs to humans, and then become established as human pathogens, comprehensive scientific studies that use the tools of ecology, virology, microbiology, and epidemiology are needed [113]. The study of disease ecology has become an established discipline with advances in both the formulation of new theory as well as the integration of molecular virological techniques that provide important information about epidemiology, ecology and viral evolution, all of which has been applied to both health and conservation [114–116]. Because ecological systems are rife with heterogeneities and often have non-intuitive processes underlying their dynamics, it is critically important for scientists to use a comprehensive approach to understanding the population processes of an ecosystem before successful intervention strategies can be developed or implemented. Admittedly this is a daunting task and it is often the case that scientists need to operate with less than complete information. Where this is the case, a modeling approach is necessary to identify key processes that allow successful interventions.

Technological advances in molecular virology and genetics, as well as the expanded use of mathematical models in epidemiology and disease ecology have dramatically changed our ability to manage both conservation and health. Finally, it is only with this type of interdisciplinary approach that considers free-ranging wildlife, domestic animals and humans as inextricable components of a single disease system, that progress can be made that will satisfy both conservation and public health needs. This is the essence of conservation medicine [117] and indeed we believe a “One Health” approach to infectious disease is necessarily the way forward.

## Supplementary Materials

Supplemental movie (Video S1).

## Figures and Tables

**Table 1.** Definitions of key terms used in the text.

**Term**	**Definition**
	**Terms from Molecular Genetics & Phylodynamics**
Admixture	The formation of a hybrid population through the mixing of two ancestral, or long-separated, populations.
Bayesian Skyline Plot	A method for estimating historical population dynamics from a sample of sequences without assuming a predefined demographic model.
Coalescent Theory	A mathematical framework which describes the distribution of gene trees in populations. It provides mathematical methods for connecting demographic or ecological models with a phylogenetic tree.
Demography	Demography is the statistical study of populations. In the field of ecology, demography encompasses the study of the size, structure and distribution of populations, and spatial and/or temporal changes in them in response to birth, migration, aging and death. However, here we use a more rigid definition of demography—as the pattern and rate of population growth.
Effective population size (Ne)	The number of breeding individuals in an idealized population that would show the same amount of dispersion of allele frequencies under random genetic drift, or the same amount of inbreeding, as the natural population under consideration. The analogue of Ne for viruses, the ‘effective number of infections’ is related to the number of infected host individuals and to the number of new transmission events [35].
Metagenomics	A discipline which uses next-generation sequencing technologies to characterize the entirety of genomic material found in environmental samples.
Molecular Clock	The molecular clock is derived from the hypothesis that sequence evolution, while random, occurs at stable rate such that the time since the divergence of two or more sequences can be estimated. Recent ‘relaxed molecular clock’ analysis can account for variation in the rate of sequence evolution through time or between lineages.
Phylogeny	The relations among a set of sequences showing which shares a most recent common ancestor with other sequences.
Phylogeography	The study of the principles and processes governing the geographical distribution of genealogical lineages.
Population Structure	In the field of population genetics, population structure is defined as the absence of random mating within a population. This is the definition used here. In ecology, population structure is defined by several key parameters including number of individuals in a population, age distribution of individuals, probabilities of survival (or mortality), and rates of fecundity.
Reassortment	A process that occurs in segmented viruses by which one or more segments ‘swap’ to create a new viral genome. This drives the process of antigenic shift in Influenza A viruses.
Recombination	The process by which new genotypes are created by the combination of distinct lineages. In sexual organisms it occurs during meiotic division, by the exchange of DNA between different chromosomes or ‘crossing-over’. Viral recombination occurs during viral replication and is an important factor in viral evolution (for more details see Worobey and Holmes, 1999 [36]).
Reversion	Mutation of a virus such that it changes ‘back’ to its wild-type state.
**Terms from Ecology**	
Life History	The timing of an organism’s schedule of reproduction and death. Species with long life histories, also known as ‘K’ strategists, tend to have low reproductive rates, stable populations, long generation times and long lifespans.
**Terms from Disease Ecology**	
Aggregation	Where the parasite population is not randomly distributed among hosts, such that the variance is greater than the mean. The macroparasites in a host population are often best described by the negative binomial distribution such that a minority of hosts possess the majority of the parasites.
R_0_ (Basic Reproduction Number)	In the case of viruses and other microparasites, R_0_ is the average number of secondary infections which an infection produces. As such it is a measure of parasite fitness.
Reservoir Host	A host species that can independently maintain a disease and act as a source of infection to other host species. Infection in reservoirs is usually more persistent and less harmful than that of other hosts.
Zoonotic disease	A disease transmissible from animals to humans or *vice versa.*
